# Preliminary study exploring the association between amygdala-ventral medial prefrontal-cortex connectivity and anxiety among adolescent and young adult cancer survivors

**DOI:** 10.1186/s12885-025-15328-w

**Published:** 2025-12-29

**Authors:** Robert Knoerl, Andrew Jahn, Katherine Grandinetti, Leslie A. Fecher, N. Lynn Henry, Yasmin Karimi, Robert Ploutz-Snyder, Scott Schuetze, Emily Walling, Alexandru Iordan

**Affiliations:** 1 University of Michigan School of Nursing, 400 North Ingalls St, Office 2350, Ann Arbor, MI 48109 USA; 2 Department of Radiology, Michigan Medicine, Ann Arbor, MI 48109 USA; 3 School of Nursing, University of Michigan, Ann Arbor, MI 48109 USA; 4 Medical Oncology, University of Michigan Rogel Cancer Center, Ann Arbor, MI 48109 USA; 5 Pediatric Hematology/Oncology, C.S. Mott Children’s Hospital, Ann Arbor, MI 48109 USA; 6 Department of Psychiatry, University of Michigan, Ann Arbor, MI 48109 USA

**Keywords:** Adolescent, Young adult, Anxiety, Oncology, Magnetic resonance imaging

## Abstract

**Background:**

Up to 40% of adolescent and young adult cancer survivors (AYAs) experience anxiety after cancer treatment. Evidence from healthy controls suggests that the prefrontal cortex exerts a regulatory influence over the amygdala, facilitating reappraisal of fear-inducing stimuli and preventing maladaptive emotional responses. Increased anxiety severity has been associated with low amygdala–ventral medial prefrontal cortex (vmPFC) connectivity, but little is known about the neural correlates of anxiety in AYA cancer survivors. The purpose of this cross-sectional study was to explore the association between amygdala-vmPFC connectivity and anxiety severity among AYA cancer survivors.

**Methods:**

Seventeen post-treatment AYA cancer survivors were recruited from the University of Michigan. Participants completed an anxiety self-report and a 45-minute functional MRI scan that consisted of resting and task-based conditions (i.e., viewing pleasant, neutral, or unpleasant scenes). General linear models were constructed to investigate the effect of self-reported anxiety on the functional connectivity between the amygdala and vmPFC regions.

**Results:**

Anxiety severity was positively associated with the functional connectivity between the right amygdala and vmPFC (MNI: -2, 54, 14; *k* = 323 voxels; peak t-statistic = 9.16; peak beta = 0.34; *p* = 0.04) while viewing pleasant images, but not other images or during rest. Analyses using the left amygdala as a seed region did not yield significant correlations.

**Conclusions:**

The positive association between anxiety severity and amygdala-vmPFC connectivity while viewing positive stimuli may suggest an overly active neural pathway linked to overall ineffective emotional regulation. Further understanding of amygdala-vmPFC connectivity as a potential mechanism underlying anxiety may provide evidence toward a viable target for intervention research.

## Introduction

Adolescent and young adult (AYA) cancer survivors (15–39 years old) are a distinct cancer survivorship subgroup characterized by differences in psychosocial development (e.g., educational attainment, career, relationships, and identify development) [1] relative to older adult or pediatric cancer survivors. Large-scale cross-sectional analyses indicate that individuals diagnosed with cancer as an AYA report the highest frequency of lifetime mental health issues, such as anxiety, in comparison to adults (>50 years old) who were diagnosed with cancer and individuals without cancer [2]. In the long term, anxiety is associated with severe fear of cancer recurrence [3, 4], work/school concerns [5, 6], fatigue [7, 8], sleep problems, and increased health care expenses [9]. Results from a meta-analysis reveal that AYA cancer survivors are at an elevated risk of developing anxiety disorders (*OR* = 1.16) in comparison to AYAs without cancer [10]. Anxiety negatively affects quality of life as AYAs report worry about meeting new people, avoiding social situations [11] and planning for the future [12]. While a variety of non-pharmacological interventions have been tested (e.g., technology- based, psychoeducational, physical exercise), there is no strong evidence to support a specific intervention to reduce anxiety in AYA cancer survivors, although AYAs desire further psychological support [6, 13]. One key barrier to the development of novel anxiety treatments in AYAs is the limited understanding of the underlying mechanisms driving anxiety in this group.

The amygdala and medial prefrontal cortex are two brain regions involved in the neuronal circuitry underlying the mechanisms of anxiety (e.g., emotional response to vague, potential threats) [14]. Evidence suggests that during normal anxiety extinction processes, the prefrontal cortex (a brain structure implicated in the regulation of emotion [15] working memory, and planning) [16] exerts control over the amygdala (a brain structure implicated in the fear response) [17], thus facilitating reappraisal of fearful stimuli and preventing maladaptive emotional responses [18]. A feature of anxiety in adults is dysregulated functional connectivity (i.e., temporal correlation between neural activity in different brain regions) [18] between the amygdala and medial prefrontal cortex [19–21]. Evidence from resting-state functional magnetic resonance (fMRI) data suggests that healthy individuals with increased self-reported anxiety have low amygdala-ventral medial prefrontal-cortex (vmPFC) connectivity, while individuals with low self-reported anxiety have greater amygdala-vmPFC connectivity [22]. Further, evidence has shown a negative correlation (*r* = −0.60) between anxiety symptoms and right amygdala-vmPFC connectivity among individuals with generalized anxiety disorder over one year [23]. However, less is known surrounding the relationship between amygdala-vmPFC connectivity and anxiety severity among AYA cancer survivors. We chose to focus on AYA cancer survivors given the increased risk for anxiety and associated reductions in quality of life in this younger age group. The identification of mechanistic targets will allow for the design of interventions to decrease anxiety in AYA cancer survivors.

The overall objective of this proof-of-concept study is to explore neurobiological mechanisms of anxiety in AYA cancer survivors. The primary aim was to explore the association between amygdala-vmPFC connectivity and anxiety severity among AYA cancer survivors using resting-state and task-based fMRI methodology.

## Materials and methods

### Design, sample, and setting

The primary aim was investigated using a prospective, cross-sectional design. AYA cancer survivors were recruited from the adult (i.e., breast, sarcoma, melanoma, leukemia, lymphoma) and pediatric oncology outpatient clinics at the University of Michigan Rogel Cancer Center and C.S. Mott Children’s Hospital. Patients were eligible if they were (1) 15–39 years old, (2) at least one month-post cancer treatment, (3) spoke/read English, and (4) completed cancer treatment within the past five years. Participants were excluded from the study if they had a prognosis of less than three months or did not meet fMRI safety guidelines (i.e., had ferromagnetic metallic or electronic implants in the body, claustrophobic or unable to lie on their back for an hour, or were pregnant). Prior to the scan, a study staff member administered the fMRI safety screening form, as required by the laboratory. All participants provided written informed consent via SignNow (airSlate, Newport Beach, CA). For individuals younger than the age of 18, parental permission for the child to participate was obtained first and then assent from the child to participate was obtained second. The study was approved and regulated by the University of Michigan Medical School Institutional Review Boards (IRBMED) (HUM00210714). Participants received a $25 gift card for completing the surveys and scan.

### Measures

Participants completed the Patient-Reported Outcomes Measurement Information System (PROMIS) Emotional Distress – Anxiety – Short Form 4a, PROMIS Emotional Distress – Depression – Short Form 4a, Perceived Stress Scale, and demographic questions prior to the fMRI scan. The PROMIS Anxiety 4a subscale measures self-reported fearfulness, worry, nervousness, and uneasiness over the past 7 days. Each item is rated on a 1–5 scale, with a “5” representing more severe anxiety [24]. Transformed total scores range from 40.3 to 81.6 (higher scores = worse anxiety). The PROMIS Anxiety 4a and Depression 4a scores were transformed using the HealthMeasures Scoring Service (calibration sample = “Cancer”). Calibration testing was conducted to determine whether PROMIS items performed differently among individuals with varying clinical conditions in comparison to the general population [25, 26] (e.g., among individuals with varying cancer diagnoses and treatments such as in the cancer calibration sample). Satisfactory convergent validity is evidenced by strong correlations between the PROMIS Anxiety 4a and the Generalized Anxiety Disorder-7 (*r* = 0.79) and the 5-Item Mental Health Inventory (*r* = 0.85) [27]. The PROMIS Depression 4a measures participants’ perceptions of mood, views of self, and affect over the past week (total scores range from 41.0 to 79.4; higher scores represent worse depression severity) [27]. The Perceived Stress Scale measures the degree to which life events are perceived as stressful over the past month (total scores range from 0 to 40; higher scores representing greater stress) [28]. After the scan, study staff abstracted cancer type and stage, cancer treatment history, comorbid conditions, and anxiety treatment.

### fMRI functional connectivity

#### Scan acquisition parameters

MRI images were acquired using a 3 Tesla GE Discovery MR750 scanner equipped with 32-channel head coil (Nova Medical). Head movement was minimized through: (a) instructions to the participant and (b) surrounding the head inside the head coil with foam padding and pillows. Stimuli were presented via a MR-compatible LCD monitor (Nordic Neurolab). Responses were recorded by a MR-compatible fiber-optic button response device (Psychology Software Tools). Task presentation and response-collection was completed using PsychoPy. Functional T_2_^*^-weighted blood oxygen level-dependent (BOLD) images were acquired using a multiband sequence of 60 contiguous axial 2.4 mm thick slices (Multiband Factor = 6, Repetition Time = 800ms, Echo Time = 30ms, flip angle = 52°, field of view = 21.6 cm, 90 × 90 matrix, 2.4 mm isotropic resolution). The contiguous slices optimize the effectiveness of the movement post-processing algorithms. The thin slice-thickness was chosen to minimize the signal voids that result from magnetic susceptibility gradients near air/tissue boundaries. After scout acquisitions to localize the head in the MRI scanner, two structural image sets were acquired: T_1_–weighted gradient echo images acquired before the functional scans using the same field of view and slices as the functional scans (Repetition time = 250ms, Echo Time = 3.7ms, flip angle = 90°); and a whole-brain, high resolution, 3D inversion recovery-prepped spoiled gradient echo images acquired after the functional scans (Inversion Time = 1060ms, flip angle = 8°, field of view = 25.6 cm, 1 mm slice thickness, 208 sagittal slices, 256 × 256 matrix, 1mm^3^ isotropic resolution).

#### fMRI Study Design

All enrolled participants were scheduled to undergo a 45-minute resting-state and task-based fMRI scan at the University of Michigan Functional MRI Laboratory. Functional connectivity between the amygdala and vmPFC was assessed during resting-state and task-based fMRI. For the resting-state fMRI scans (two 6-minute runs for 12 min total resting-state) [29], participants were instructed to view a fixation cross in the center of the screen while keeping their mind calm and relaxed. For the task-based fMRI scan, participants were presented with 36 unpleasant/emotionally negative (e.g. snake or ship sinking), 36 neutral (e.g., shoes or table), and 18 pleasant/emotionally positive (e.g., people smiling) scenes, selected from the International Affective Picture System [30]. A full list of the specific International Affective Picture System image IDs used in this study are provided in Supplementary Table S1. To ensure participants were paying attention to the stimuli, the participants were asked to respond whether each stimulus depicted an indoor or outdoor scene via button press. Scenes were presented in blocks of six consecutive stimuli, with each stimulus displayed for four seconds. Within each block, stimuli had similar emotional valence (i.e., negative, positive, or neutral), with indoor/outdoor scenes displayed in random order. Scene blocks (24 s duration) were separated by 12 s rest blocks, when only a fixation cross was displayed, to allow for the hemodynamic response to return to baseline. Each scanning run was comprised of two negative blocks, two neutral blocks, and one positive block, alternating pseudo-randomly. Resting-state recording preceded task-based fMRI to avoid carry-over effects. Effects of motion were mitigated by censoring scans with a differential displacement *d* >0.5 mm or global intensity *z* >3 using linear regression. Participants were monitored for respiration and oxygen levels during the scan. Cardiac and respiratory noise correction was performed using RETROICOR [31].

#### Preprocessing and 1 st -Level Analysis

Both the resting-state and task-related functional data were analyzed using the CONN toolbox, version 22a [32]. The default-preprocessing pipeline was used, which consisted of slice-timing correction, motion correction, coregistration of the functional and anatomical images, normalization to MNI space, and smoothing using an 8 mm kernel. For denoising, anatCompCor was applied, which uses principal components analysis to extract the top five components from the white matter and cerebrospinal fluid, and then regress them from the grey matter mask. Images were inspected for data quality assurance after preprocessing, which included visualizing the fit of coregistration and normalization between the functional images and the MNI template, as well as identifying any subjects that had more than 10% of their functional scans removed due to being above our motion threshold of *d* >0.5 mm. No subjects were excluded based on these checks.

Amygdala-vmPFC connectivity was assessed during both resting-state and task-performance. To identify the amygdala, we employed the SPM Anatomy Toolbox [33] which provides probabilistic maps for the entire amygdala and its subnuclei. We defined the left and right amygdalae as two separate seed regions and calculated seed-to-voxel connectivity targeting the vmPFC (cluster-forming threshold of *p* = 0.01, with a cluster-based false discovery rate [FDR] corrected at *p* < 0.05). Average time-series for each region (e.g., left and right amygdalae) were extracted separately for each condition of interest (e.g., negative and neutral) and time point (i.e., pre- and post-intervention), correlated with each voxels’ time series, and *z*-transformed using the Functional Connectivity Toolbox (CONN).

### Statistical analyses

To investigate the modulation of self-reported anxiety (i.e., PROMIS Anxiety 4a) on the functional connectivity of the amygdala, we created a general linear model that included effects of rest and affect (positive, neutral, negative) for the resting-state and task-based scan, respectively, while controlling for sex and age. All covariates were demeaned. Motion parameters estimated during motion correction were inserted into the model as regressors of no interest, as were individual regressors identifying volumes that exceeded *d* > 0.5 mm. Statistical significance of the results was determined using the thresholds described above (Preprocessing and 1 st -Level Analysis).

## Results

### Participant characteristics

**Fig. 1:**
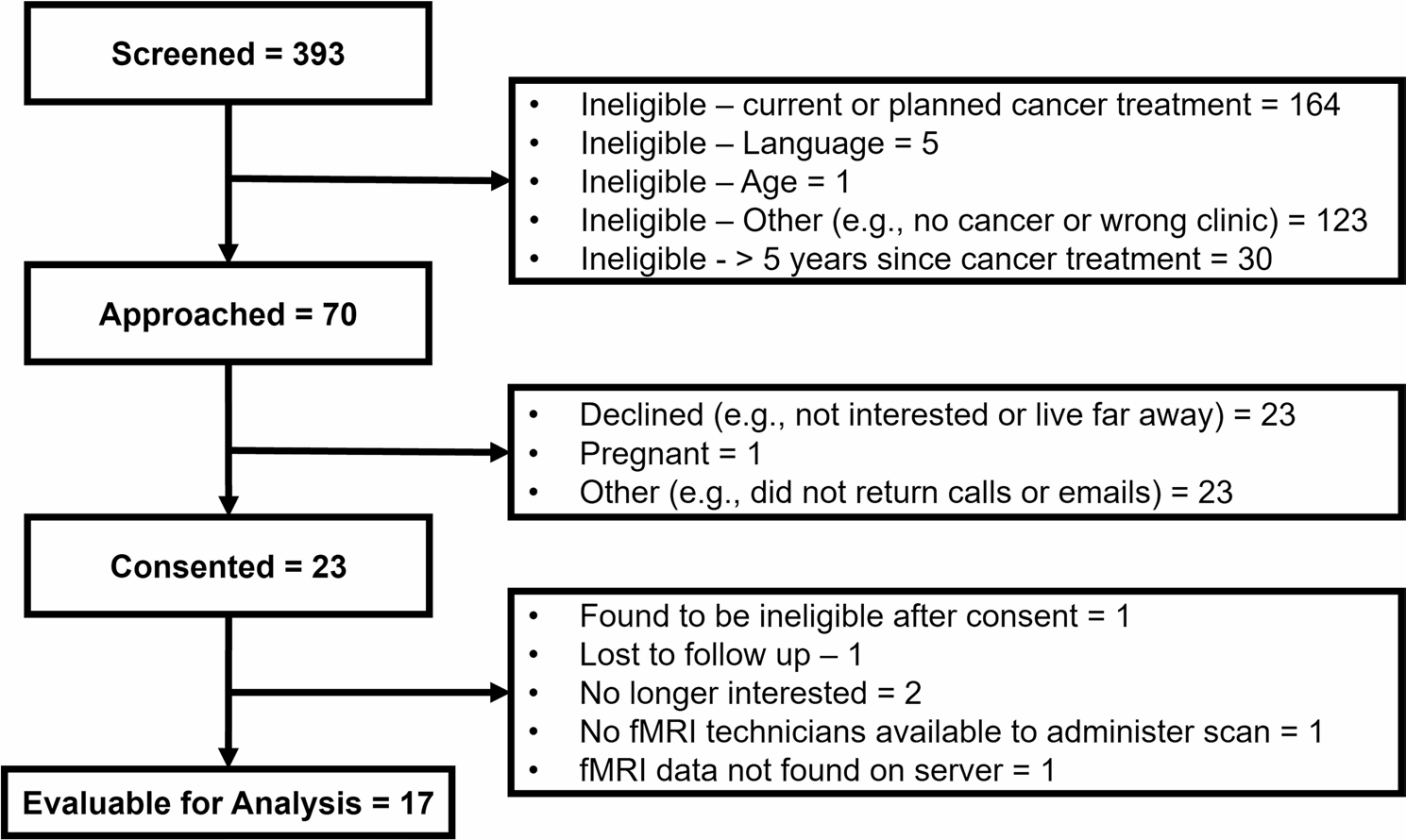
Participant Flow Study Diagram. Figure 1 describes participant flow through the study

Figure 1 describes participant flow through the study. Study recruitment and data collection occurred from 5/16/2022 to 11/28/2022. Table 1 describes the demographic characteristics of the participants at baseline. On average, participants (*N* = 17) were 27.29 years old (*SD* = 7.9, *Range* = 15–39) and there was an average of 528.88 days between the end of cancer treatment and the time of the fMRI scan (*SD* = 371, *Range* = 42–1655). Participants were mainly male (64.7%), white (100%), single (70.6%), and diagnosed with leukemia (41.2%), lymphoma (29.4%), Ewing sarcoma (17.6%), melanoma (5.9%), and breast cancer (5.9%). Participants’ median scores on the PROMIS Anxiety 4a, PROMIS Depression 4a, and Perceived Stress Scale were 52.3 (*Range* = 40.5–67.5), 48.9 (*Range* = 41–58.9), and 15 (*Range* = 5–27), respectively. Overall, 41% of the sample reported PROMIS Anxiety 4a scores at a mild (scores ≥ 55) or greater severity level [33].

**Table 1:** Participant characteristics at baseline (*N* = 17)

Characteristics	Frequency (*n*, %)
Age	
15–17 years old	2 (11.8%)
18–25 years old	6 (35.3%)
26–39 years old	9 (52.9%)
Sex	
Male	11 (64.7%)
Female	6 (35.3%)
Race	
White	17 (100%)
Ethnicity	
Not Hispanic or Latino	17 (100%)
Education	
Some high school	2 (11.8%)
Some college or technical training	5 (29.4%)
University undergraduate degree	6 (35.3%)
University post graduate degree	4 (23.5%)
Marital Status	
Single	12 (70.6%)
Married	5 (29.4%)
Employment Status	
Working full time	7 (41.2%)
Working part-time	2 (11.8%)
Student^a^	5 (29.4%)
Not working	3 (17.6%)
Cancer Type	
Leukemia	7 (41.2%)
Lymphoma	5 (29.4%)
Ewing Sarcoma, Localized	3 (17.6%)
Melanoma	1 (5.9%)
Breast	1 (5.9%)
Cancer Stage^b^	
Stage I	0
Stage II	2 (28.6%)
Stage III	0
Metastatic	4 (57.1%)
Missing/Unknown	1 (14.3%)
Treatment Type ^c^	
Chemotherapy	16 (94.1%)
Immunotherapy	2 (11.8%)
Surgery	3 (17.6%)
Radiation	2 (11.8%)
Hormone Therapy	0
Other^d^	2 (11.8%)
Currently receiving anxiety or depression medications	
Yes	7 (41.2%)
No	10 (58.8%)
Comorbid Psychological Conditions	
Yes	9 (52.9%)
No	8 (47.1%)
Mean Patient Reported Outcome Scores ( ***SD*** )	
PROMIS Anxiety 4a	54.35 (8.86)
PROMIS Depression 4a	49.92 (6.86)
Perceived Stress Scale	14.35 (6.08)

^a^ One participant selected “Working part-time” and “Student.” The participant’s selection was counted as student given participant was less than 18 years old

^b^ Reflects disease severity for participants with breast cancer, melanoma, or lymphoma (*n* = 7)

^c^ Total sums to greater than 100% as participants may have been receiving more than one type of cancer treatment

^d^ Two participants received allogenic stem cell transplants

### Modulation of patient-reported anxiety on the functional connectivity of the amygdala

**Fig. 2:**
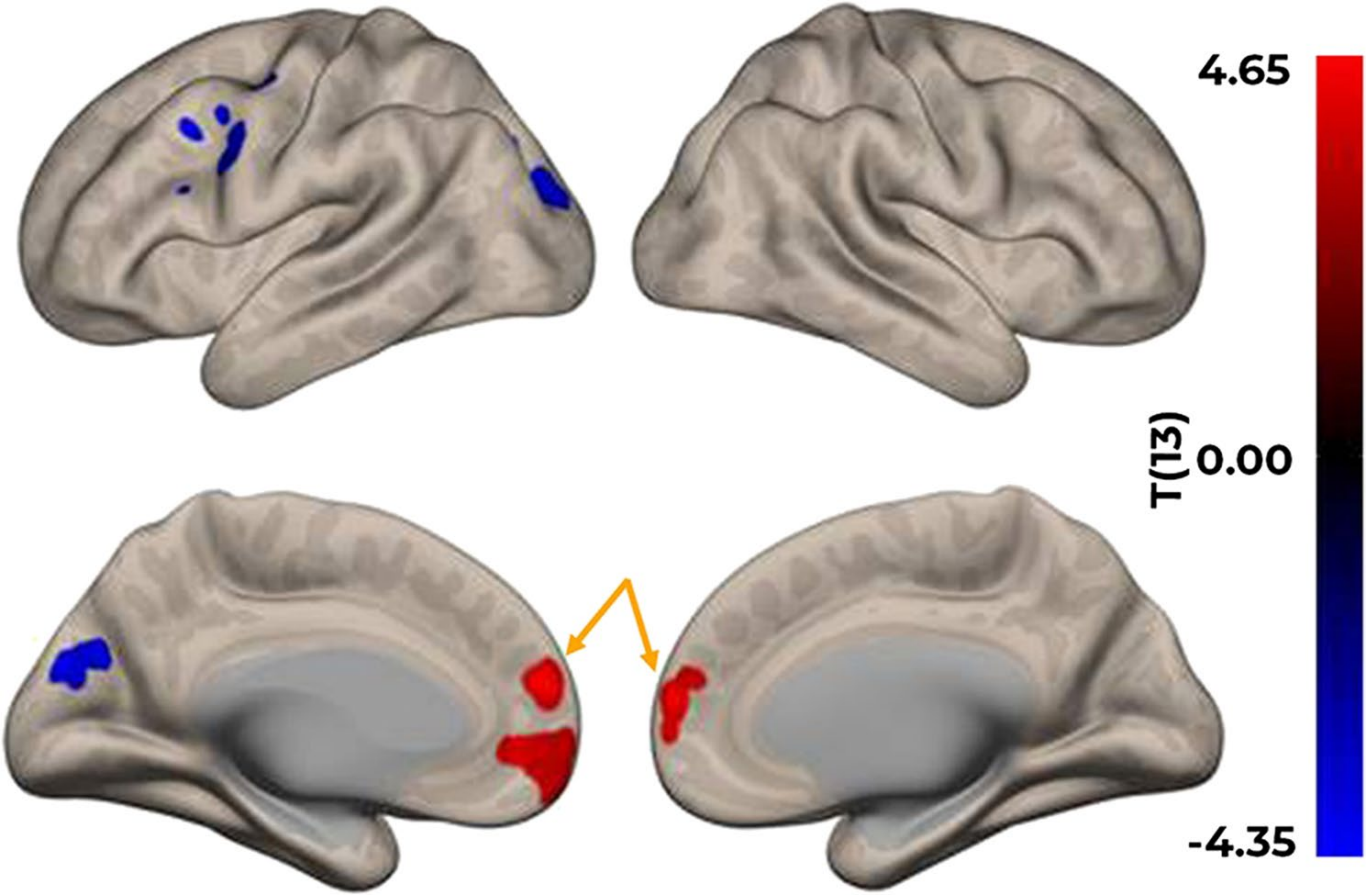
Connectivity results using the right amygdala as a seed region, thresholded at CDT of *p* = 0.01, FWE < 0.05. Significantly positive correlation was observed between measurements of anxiety severity and the functional connectivity between the right amygdala and the rostral and ventral MPFC regions (indicated by orange arrows)

Using the right amygdala as a seed region, anxiety severity was positively correlated with the functional connectivity between the amygdala and the vmPFC (MNI: −2, 54, 14; *k* = 323 voxels; peak t-statistic = 9.16; peak beta = 0.34; *p* = 0.04) while participants were viewing pleasant scenes (i.e., the positive condition; see Fig. 2). No other significant findings were found between anxiety severity and right-amygdala-vmPFC connectivity for the neutral or negative task-based conditions or resting-state scans. Analyses using the left amygdala as a seed region did not yield any significant correlations with either the vmPFC or other regions of the brain, using the statistical thresholds described above for the resting-state or task-based scans.

## Discussion

Study results showed that anxiety severity was positively associated with functional connectivity between the right amygdala and the vmPFC while viewing pleasant scenes. Our findings are inconsistent with prior research demonstrating that higher anxiety is associated with lower connectivity between the amygdala and vmPFC during rest [22]. While the vmPFC has been traditionally associated with extinction of fear responses and modulation of negative emotions through inhibitory control over the amygdala [17, 18], emerging views suggest that the vmPFC (Brodmann Area 10/32) plays a more nuanced role, including modulation of emotional arousal, self-awareness, and self-referential processing [34–38], with some evidence pointing to an overall affinity for positive affect [39, 40]. Thus, our findings suggest disruption of the vmPFC’s ability to integrate and/or modulate emotional arousal in AYA cancer survivors with increased anxiety.

Furthermore, in line with the “valence hypothesis,” the left hemisphere of the brain is posited to be more specialized for processing positive emotions, whereas the right hemisphere is more specialized for processing negative emotions [41]. This pattern has been proposed to also extend to the amygdala [42]. Therefore, our findings showing greater right amygdala-vmPFC coupling for those with higher anxiety further strengthen the idea of abnormal engagement of emotion processing mechanisms in AYA cancer survivors. In sum, the positive association between anxiety scores and amygdala-vmPFC functional connectivity may represent a maladaptive engagement of emotion processing and integration/regulation mechanisms, where heightened amygdala reactivity may drive greater vmPFC engagement to regulate emotional arousal while viewing pleasant scenes. Future research with a larger sample of AYA cancer survivors is needed to confirm our findings and to investigate differences by age, durations since diagnosis or treatment, concomitant medication use, and stage of disease.

Further understanding of amygdala-vmPFC connectivity as a potential mechanism underlying anxiety in AYA cancer survivors will allow for scientists to target interventions for AYA cancer survivors with anxiety based on this mechanism. For example, music-based interventions may be one possible intervention to improve amygdala-vmPFC connectivity among AYA cancer survivors with anxiety. The amygdala is a potential physiological target of music-based interventions for anxiety because the basolateral amygdala receives direct input from the auditory cortex (i.e., to perceive the positive or negative value of music) [43] and the medial prefrontal cortex [44]. Prior evidence suggests that activity in various brain structures, including the amygdala and PFC, is altered in response to music [43, 45]. Consistent with the neural bases of other behavioral treatments for anxiety (e.g., cognitive-behavioral therapy or mindfulness-based stress reduction) [46–48], music therapy may exert top-down vmPFC control over the amygdala. However, no studies have evaluated the impact of music-based interventions on changes in amygdala- vmPFC connectivity.

### Limitations

There are several limitations to this analysis. A major limitation of our study is the absence of a concurrently recruited non-cancer control group assessed using identical measures and scanning protocols. As a result, our findings cannot directly address whether patterns of amygdala-vmPFC connectivity in AYA cancer survivors differ from those in the general population. The external generalizability of the results is limited given the sample was homogenous with regard to race, ethnicity, and institution. The sample size was small and therefore employed a less stringent cluster-forming threshold for statistical significance (*p* < 0.01) than what is typically used in fMRI experiments (i.e., a cluster-forming threshold of *p* < 0.001) [49, 50]. Anxiety was measured using the PROMIS Anxiety 4a immediately prior to the fMRI scan, yet, it is possible that participants’ anxiety level was modified in response to the scanning environment (e.g., scanner is loud and may feel claustrophobic) or other variables that were not measured or controlled for in the analysis (e.g., co-occurring fatigue, insomnia, or pain [51], anxiety medication use, comorbid psychological conditions, cancer type, or time since cancer treatment). Similarly, there was not a sufficient sample size to explore potential differences in the relationship between anxiety and amygdala-vmPFC connectivity among AYAs of different developmental spectrums (i.e., adolescents [15–17 years], emerging adults [18–25 years], young adults [26–39 years]) [52]. Lastly, the analysis solely focused on the relationship between anxiety and amygdala-vmPFC connectivity, but there are many other brain structures and processes implicated in the development of anxiety [15].

## Conclusions

Right amygdala-vmPFC functional connectivity was positively associated with patient-reported anxiety severity among a sample of AYA cancer survivors during the positive task-based condition, but not during the neutral or negative conditions or resting state. Further studies are needed to replicate and confirm the directionality of the relationship between amygdala-vmPFC connectivity and anxiety severity among AYA cancer survivors. Such mechanistic findings may provide evidence toward a viable target for intervention research (e.g., music therapy) for the management of anxiety in AYAs and other cancer populations (e.g., older adults), which will be highly impactful given that ~ 18% of cancer survivors have an anxiety disorder [53].

## Data Availability

The datasets used and/or analyzed during the current study are available from the corresponding author on reasonable request.

## Abbreviations

AYA: Adolescent and young adult
fMRI: Functional magnetic resonance imaging
PROMIS: Patient-Reported Outcomes Measurement Information System
vmPFC: Ventral medial prefrontal cortex
